# The Role of Histone Acetyltransferases in Normal and Malignant Hematopoiesis

**DOI:** 10.3389/fonc.2015.00108

**Published:** 2015-05-26

**Authors:** Xiao-Jian Sun, Na Man, Yurong Tan, Stephen D. Nimer, Lan Wang

**Affiliations:** ^1^Sylvester Comprehensive Cancer Center, University of Miami Miller School of Medicine, Miami, FL, USA; ^2^Department of Cell Biology, University of Miami Miller School of Medicine, Miami, FL, USA; ^3^Department of Biochemistry and Molecular Biology, University of Miami Miller School of Medicine, Miami, FL, USA; ^4^Department of Medicine, University of Miami Miller School of Medicine, Miami, FL, USA

**Keywords:** histone acetyltransferases, hematopoiesis, transcriptional regulation, hematopoietic stem cells, hematological malignancies

## Abstract

Histone, and non-histone, protein acetylation plays an important role in a variety of cellular events, including the normal and abnormal development of blood cells, by changing the epigenetic status of chromatin and regulating non-histone protein function. Histone acetyltransferases (HATs), which are the enzymes responsible for histone and non-histone protein acetylation, contain p300/CBP, MYST, and GNAT family members. HATs are not only protein modifiers and epigenetic factors but also critical regulators of cell development and carcinogenesis. Here, we will review the function of HATs such as p300/CBP, Tip60, MOZ/MORF, and GCN5/PCAF in normal hematopoiesis and the pathogenesis of hematological malignancies. The inhibitors that have been developed to target HATs will also be reviewed here. Understanding the roles of HATs in normal/malignant hematopoiesis will provide the potential therapeutic targets for the hematological malignancies.

## Introduction

Histone acetyltransferases (HATs) acetylate histone proteins by transferring acetyl group from acetyl-CoA to specific lysine residues (1, 2). The acetylation of histones by HATs results in a dispersed structure of chromatin, which becomes accessible by transcriptional factors. Besides histones, a variety of non-histone substrates also have been shown to be acetylated by HATs, thus the HATs are now generally categorized as lysine acetyltransferases (3). The acetylome studies have led to discovery of many new substrates of HATs, and a lot of non-histone substrates of HATs, such as AML1, AML1-ETO (AE), p53, c-MYC, NF-κB, Cohesin and Tubulin, have been found to play important roles in different cellular processes (4–10). Based on the cellular localization, HATs are classified into type A and type B HATs. The type A HATs show nuclear localization and likely catalyze the processes related to transcription (11). The type A HATs are further divided into five families according to their homology and acetylation mechanisms. The GNAT family members include PCAF, Gnc5 and ELP3. CBP and p300 form the CBP/p300 family (12). Tip60, MOZ, MORF, HBO1 and HMOF belong to the MYST family (13). The transcriptional factor related HAT family includes TAF1 and TIFIIIC90. In addition, several steroid receptor co-activators, such as p600, SRC1, CLOCK and AIB1/ACTR/SCR3 etc., are also HATs. (14, 15). Type B HATs are localized in the cytoplasm and they are shown to acetylate the newly synthesized histones. For example, HAT1 is one of type B HAT members and functions in DNA repair and histone deposition (16).

**Figure 1:**
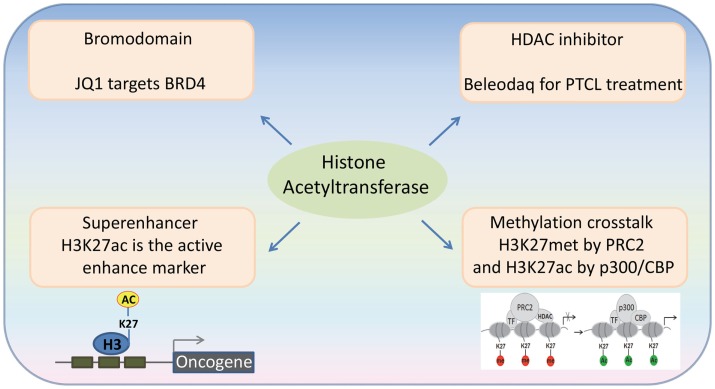
**Recent advances in the emerging fields of histone acetylation in hematopoiesis: (1) Bromodomains are a promising target for the therapy of hematological malignancies; (2) HATs generate histone marks found in active enhancers; (3) Histone acetylation-methylation crosstalk in hematopoiesis; (4) Third generation HDAC inhibitors for the therapy of hematological malignancies**.

Histone acetyltransferases play key roles in normal and malignant hematopoiesis. The acetylation of histones and non-histone proteins has been shown to regulate normal blood cell development (17–19) (Table 1). Analysis of chromatin factor interaction network in hematopoietic development shows multiple chromatin factor complexes, including NuA4/P300/CBP/HBO1, are required for normal hematopoiesis (20). Protein acetylation regulates hematopoietic stem cell (HSC) self-renewal, proliferation, and their differentiation into committed hematopoietic progenitors. In line with the critical functions of HATs in normal hematopoiesis, chromosomal translocations that involve HAT genes are frequently found in hematological malignancies. Recent cancer genome studies have identified HATs as common targets for mutations in these diseases. Meanwhile, the acetylation states of some oncoproteins and tumor suppressor proteins have been correlated with hematological malignancies manifestation (5, 19, 21–25) (Table 2). Notably, most leukemogenic fusion proteins physically interact with HATs, even though they are not directly fused with HATs, suggesting that the aberrant acetylation regulation by these fusion proteins are critically important in leukemogenesis (26). In this article, we will first briefly review a few examples of interesting findings that potentially lead to development of new therapeutic strategies for hematological malignancies, and then provide an overview of the functions of HATs in normal and malignant hematopoiesis (Figure 1).

**Table 1:** **The role of HATs in hematopoietic stem/progenitor cells**.

Cell type	Acetyl-transferase	Target genes	Established role/function
Hematopoietic stem/progenitor cells	p300	C-Myb	Block proliferation and promote differentiation
	CBP	Gfi1b	Promote self-renewal and block differentiation
	MOZ	p16	Generate and maintain HSCs
Myeloid progenitor cells	p300/CBP	C-Myb	Block proliferation and promote differentiation
	MOZ	p16	Promote hematopoietic progenitors proliferation
	HBO1	Gata1	Promote fetal liver erythropoiesis
Lymphoid cells	p300	Foxp3/C-Myb	Regulate Foxp3(+) Treg cell function and homeostasis
	GCN5	PI3K/AKT/Syk/Btk	Regulate B cell apoptosis

**Table 2:** **The role of HATs in hematological malignancies**.

Acetyltransferase	Disease	Non-histone substrate	Target genes	Established role/function	Inhibitors
p300/CBP	AML	C-Myb, AML1–ETO	Id1, p21, Egr1	Block differentiation and promote self-renewal	C646, EGCG, L002
p300	T cell leukemia	Notch3	Unknown	Promote Notch3-induced T cell proliferation	Garcinol
Tip60	AML, lymphoma	Unknown	C-Myc, p53	Tumor suppressor and modulate DDR signaling	Garcinol
MOZ/MORF	AML	AML1	p53, RARβ, PU.1	MOZ-related fusion proteins transduce HSPCs	Garcinol
GCN5	B cell ALL, AML	E2A-PBX1, AME	Syk, Btk	Promote cell transformation	MB-3, Garcinol
PCAF	AML, CML	AME	AML1	Promote AML1-dependent transcription	MG153, Garcinol

## Bromodomains are Promising Targets for Treating Hematological Malignancies

**Figure 2:**
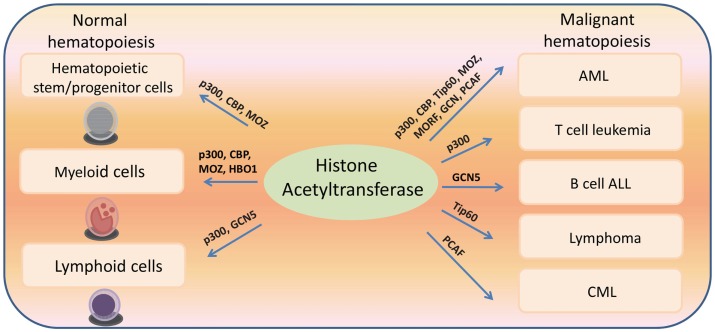
**Histone acetyltransferases regulate both normal and malignant hematopoiesis**.

Acetylated lysine residues generated by HATs can be specifically bound by some protein domains (“readers”). Bromodomains have been identified as an important type of the readers of acetyl lysine. The human genome encodes over 60 bromodomain proteins, including HATs, HAT-associated proteins (such as GCN5L2, PCAF, and BRD9), histone methyltransferases (such as ASH1L and MLL), transcriptional co-activators (such as TRIMs and TAFs), as well as the BET family proteins (27). BRD4, a member of the BET family proteins, is shown to locate at the enhancer and/or promoter regions of many active genes. The bromodomain inhibitor JQ1 is able to disassociate BRD4 from acetylated histones, leading to downregulation of gene transcription and decreased phosphorylation of RNA polymerase II (28) (Figure 2). JQ1 is also able to remove BRD4 from the super-enhancers and thus repress many super-enhancer regulated oncogenes. These findings have been verified in hematological malignancies, such as diffuse large B cell lymphoma (DLBCL), and provide a basis for using JQ1, as well as other BET bromodomain inhibitors, in the treatment of hematological malignancies (29–31).

## HATs Generate the Markers of Super-Enhancers in Hematological Malignancies

Super-enhancers are clusters of active enhancers bound by master transcription factors and cofactors, including the mediator complex, and can promote the high-level expression of genes that control cell identity (32, 33). Cancer cells can acquire super-enhancers to active oncogenes, which suggest that super-enhancer-associated genes could be candidate oncogenic drivers (29, 34–36). Histone H3 lysine 27 acetylation (H3K27ac) by p300/CBP is used as an active enhancer mark, which biophysically facilitates opening of chromatin and recruits the co-activators that recognize ϵ-acetyl lysine through bromodomain (37). BRD4 is most commonly associated with enhancer regions, defined by the presence of H3K27ac by p300/CBP and the absence of H3K4me3. In a genome-wide study of DLBCL, a rank ordering of the enhancer regions by H3K27ac enrichment reveals that BRD4 binds to the majority of active enhancers and that the genome-wide correlation between BRD4 occupancy and H3K27ac is extremely strong. Importantly, BRD4 is highly loaded at the super-enhancers (29). These findings suggest that the HAT-mediated H3K27ac and the BRD4 recruitment may play an important role in the formation and function of the super-enhancers.

## Histone Acetylation-Methylation Crosstalk in Hematological Malignancies

Polycomb group (PcG) proteins are transcriptional repressors, and their abnormal expression is frequently associated with hematological malignancies. The PcG proteins can induce transcription repression through disassociating HATs from their target genes (38). The Polycomb repressive complex 2 (PRC2) catalyzes trimethylation of H3K27, and this activity is important for transcriptional repression. Depletion of PRC2 leads to a global increase of H3K27 acetylation, which is catalyzed by p300 and CBP. In MLL-AF9-transduced HSCs, the transcriptional activation of PcG-targeted genes has been found to correlate with a methylation-to-acetylation change (Figure 1). These findings suggest that the acetylation–methylation crosstalk plays an important role in hematological malignancies.

## The Role of Histone Acetyltransferases in Normal Hematopoiesis

### The role of HATs in hematopoietic stem/progenitor cells

#### p300/CBP Knockout Mouse studies

The initial studies showed that CBP, instead of p300, is pivotal for the self-renewal of HSCs. However, it has been shown that p300, but not CBP, is essential for hematopoietic differentiation (39). However, it has been recently reported that CBP regulates both self-renewal and differentiation in adult HSCs and that loss of CBP leads to an increase in apoptosis, differentiation, and quiescence in HSCs through regulating Gfi1b (40). The numbers of colony-forming cells and erythroid cells are reduced in mouse embryos expressing the truncated CBP protein (1–1084 amino acids), which suggests that CBP mutations disrupt primitive hematopoiesis. Abnormal endothelial precursors have been found when the CBP mutant para-aortic splanchnopleural mesoderm was cultured with stromal cells, suggesting defects in the hematopoietic microenvironment (39). *CBP*^+/−^ mice develop highly penetrant, multilineage defects in hematopoietic differentiation (41). These findings indicate that CBP has important functions in normal hematopoiesis. No such pathology was observed in *p300*^+/−^ mice. Thus, p300 and CBP play essential but distinct roles in maintaining normal hematopoiesis (42).

#### MOZ is Crucial for the Generation and Development of HSPCs

MOZ contains two coactivation domains and a HAT catalytic domain. The fetal liver cells with *moz* mutation were able to help reconstitute the hematopoietic system in the lethally irradiated recipient mice. In the Moz-deficient mice, the number of hematopoietic progenitors in all lineages was reduced, and defects in HSCs were found (43). Loss of MOZ HAT activity causes abnormalities in hematopoietic stem/progenitor cell (HSPC) numbers in mice since HSPCs lacking MOZ HAT activity cannot expand. Loss of MOZ HAT activity also leads to the disruption of B cell development in mice. MOZ-mediated acetylation has been found to play an important role, controlling the balance between differentiation and proliferation in normal hematopoiesis (26, 44). MOZ controls the proliferation of HSCs at least in part by repressing the transcription of p16. The expression level of p16 is increased in HSPCs without MOZ HAT activity, which can induce the senescence of HSPCs. Loss of p16 rescues the proliferative abnormality in the hematopoietic progenitors lacking the MOZ HAT activity. These findings indicate an important role of MOZ HAT activity in the transcription of p16 and HSPC senescence (45). Together, MOZ is essential for a fundamental property of HSCs and the development of hematopoietic progenitors.

### The role of HATs in myeloid progenitors and differentiation

#### The KIX Domains of p300/CBP are Required for Definitive Hematopoiesis

The KIX domains in p300 and CBP are responsible for interacting with other proteins, and they regulate c-Myb-mediated transcription activation and repression. Loss of the CH1 or KIX domain in p300 leads to profound abnormalities in hematopoiesis, while deletion of other portions of p300 only affects some specific lineages (46). Certain site specific point mutations in the KIX domain of p300 can disrupt the interaction between p300 and CREB/c-Myb, and mice homozygous for these mutations have many hematopoietic defects, such as anemia, thrombocytosis, megakaryocytosis, thymic hypoplasia, and B cell deficiency. However, no defects are detected in mice carrying the same point mutations in CBP. The interaction between the KIX domain of p300 and c-Myb is important for the function and development of megakaryocytes, and a synergistic genetic interaction has been found between the mutations in the KIX domain of p300 and mutations in c-Myb. CBP KIX domain mutations affect platelets, B cells, T cells, and red cells. Therefore, the KIX domains in p300 and CBP have their unique functions in normal hematopoiesis (47). Altogether, the KIX domains in p300 and CBP are essential for the normal hematopoiesis through regulating c-Myb-mediated transcription activation and repression (48).

#### The Hbo1-Brd1/Brpf2 Complex is Required for Fetal Liver Erythropoiesis

HBO1 is responsible for the acetylation of histone H4K5, K8, and K12. The interaction between ING4 and histone H3K4me3 augments the ability of HBO1 to acetylate histone H3 (49, 50). HBO1 and BRD1 can form a HAT complex and control erythropoiesis. Loss of Brd1 leads to severe anemia in mouse embryos due to abnormal erythropoiesis in the fetal liver. HBO1 and BRD1 are found to mostly co-localize in the erythroblast genome, and regulate critical developmental genes. Loss of Brd1 or depletion of Hbo1 significantly decreases the levels of H3K14 acetylation in erythroblasts. Loss of Brd1 leads to reduced expression of Gata1, the key erythroid developmental regulator, and the forced expression of Gata1 can partially rescue the abnormal erythropoiesis induced by loss of Brd1. Taken together, the Brd1–Hbo1 HAT complex is an important H3K14 HAT, which is essential for the transcriptional activation of key erythroid regulators (17).

### The role of HATs in lymphoid cells

#### p300 is Critical for the Function and Homeostasis of Foxp3(+) Treg Cells

Forkhead box P3 (Foxp3) is acetylated by p300 and is essential for the development of a Treg suppressor phenotype. Hyperacetylation of Foxp3 prevents its ubiquitination and proteasome mediated degradation, which leads to a significant increase in the Foxp3 protein level. Foxp3 acetylation can rapidly control Foxp3 protein levels in T cells, which provides a new mechanism for regulating the number and function of Treg cells (51). In the presence of a p300 inhibitor, Garcinol, p300 becomes disassociated from the FOXP3 protein complex, and subsequently FOXP3 is degraded through the lysosome-dependent system. A subset of four lysine residues, which together control the total acetylation of FOXP3, could also be acetylated by p300 (52, 53). The conditional deletion or pharmacologic inhibition of p300, was able to increase apoptosis induced by the T cell receptor in Foxp3(+) Treg cells, and inhibit tumor growth in immunodeficient mice. Together, p300 is critical for the function and homeostasis of Foxp3(+) Treg cells, and thus p300 inhibitors are able to impair the function of Treg cells without affecting T effector cells suggesting a new approach for cancer immunotherapy (54).

#### The Role of GCN5 in Lymphoid Cells

GCN5 controls the PI3K/AKT pathway activation through regulating the transcription of Btk and Syk, which are involved in PI3K/AKT pathway activation in B cells under oxidative stress. GCN5 deficiency significantly induced apoptosis in chicken DT40 cells treated with hydrogen peroxide. GCN5 is localized at the proximal 5′-upstream regions of Btk and Syk, and the expression levels of Syk and Btk were significantly decreased in GCN5-deficient chicken DT40 cells exposed to exogenous hydrogen peroxide. Moreover, the phosphorylation level of AKT was also significantly decreased in hydrogen peroxide-treated GCN5-deficient chicken DT40 cells. Together, GCN5 regulates the transcription of Btk and Syk, and is crucial for the epigenetic regulation of the PI3K/AKT pathway activation in lymphoid cells under oxidative stress (55).

## The Role of Histone Acetyltransferases in Malignant Hematopoiesis

### p300/CBP

#### The Interaction of p300/CBP with c-Myb is Required for the Induction of Acute Myeloid Leukemia

CBP/p300 is an essential co-activator for the transforming capacity of c-Myb (56). CBP/p300 is required for the ability of c-Myb to repress several key target genes involved in myeloid differentiation and p300 is recruited to c-Myb-binding sites close to c-Myb target genes (57). The interaction of p300/CBP with c-Myb is essential for leukemic transformation by the myeloid leukemia oncogenes AE, MLL-ENL, and MLL-AF9 (58). The p300–c-Myb interaction is essential for the ability of AE, MLL-ENL, and MLL-AF9 fusion proteins to confer self-renewal properties on myeloid progenitor cells. In the absence of this interaction, these fusion oncoproteins are unable to impose a block of differentiation, leading instead to terminal differentiation. Myeloid progenitors from Plt6 mice, which have a mutation in p300, are also refractory to transformation by the AE and MLL fusion proteins. Taken together, the specific interaction between p300 and c-Myb is required to control a transcriptional program, which is essential for the acquisition of self-renewal and possibly other leukemogenic properties upon expression of fusion oncoproteins. Thus, disruption of the p300–c-Myb interaction could be a potential therapeutic strategy for acute myeloid leukemia (AML) (58).

#### The Acetylation of Notch 3 by p300 in T Cell Leukemia

Notch3 is acetylated at lysine 1692 and lysine 1731 by p300, which can be deacetylated by HDAC1. The acetylation of Notch3 by p300 is able to promote its ubiquitination and protein degradation through proteasome system. Consequently, the expression level of Notch3 and its transcriptional activity are decreased in the non-acetylatable Notch3 mutant transgenic mice, which leads to defects in the Notch3 downstream signaling. The non-acetylatable Notch3 mutant can enhance Notch3-induced growth of T cell leukemia proliferation, which can be blocked by a histone deacetylases (HDAC) inhibitor. In the Notch3 transgenic mouse model, HDAC inhibitor-mediated hyperacetylation of Notch3 inhibits the proliferation of T cell leukemia/lymphoma cells. Altogether, targeting Notch3 deacetylation could be a promising therapeutic strategy for T-cell leukemia (25).

#### The HAT Domain and Bromodomain are Required for MLL–CBP-Induced Transformation in AML

The CBP gene is fused with the MLL gene in patients with *t*(11;16) MDS; the MLL–CBP fusion contains a mostly intact CBP, suggesting involvement of CBP in leukemogenesis (59). Both the CBP HAT domain and bromodomain are required for MLL–CBP-induced transformation in AML, which is usually preceded by an MDS phase. The replacement of the MLL–CBP HAT domain with the PCAF/GCN5 HAT domain enhanced the proliferation of hematopoietic progenitor cells and led to the loss of myeloid cell surface markers in these cells. These phenotypes were not observed when the CBP bomodomain of MLL–CBP was replaced by the PCAF/TAFII250 bromodomain. The recipient mice transplanted with domain-swapped hematopoietic progenitors developed lymphoid disease or had low-frequency MDS that progressed to AML. Thus, the CBP HAT domain and bromodomain have different functions but play important roles in the pathogenesis of MLL–CBP-positive leukemias (60).

#### The Acetylation of the AE Fusion Protein by p300 is Required for the Induction of Acute Leukemia

We have shown that transcriptional activation by AE is crucial for leukemogenesis and that AE interacts with the transcriptional co-activator, p300. The important function of the AML1–ETO/p300 interaction is that AE can be acetylated, as acetylation of AE is essential for its self-renewal promoting effects. The acetylation of AE9a by p300 at a specific lysine residue (K43) is required for its ability to induce leukemia in mice. Pharmacological and RNA interference-mediated inhibition of p300 specifically impairs AE/AE9a-induced transcriptional activation and leukemogenesis, but does not affect the development of MLL–AF9-induced leukemia. Acetyl-AEK43 is present in blast cells isolated from *t*(8;21) leukemia patients and these leukemia cells, but not normal human CD34^+^ cells, are sensitive to growth inhibition by p300 inhibitors. AE and p300 co-localize at the regulatory regions of many AE upregulated genes, which includes the regulators of self-renewal (e.g., *Id1*, *p21*, and *Egr1*). AE and p300 can cooperate in the transcriptional regulation of these target genes. Several TFIID subunits that specifically bind to a K43 acetylated AML1 peptide but not to the identical non-acetylated peptide. Furthermore, these results establish a novel link between post-translational modification of a non-histone protein (by a “histone modifying enzyme”) and transcriptional regulation, which has crucial implications for the study of cancer and the regulation of gene expression. As ETO is thought to be a key component of co-repressor complexes, its interactions with the co-activator p300 in cells is surprising and raises a question about whether “bipotential” complexes, like bivalent histone marks, may allow cells to turn on or off a given set of genes in response to certain internal or external signals. Thus, acetylation of an oncogenic fusion transcription factor itself can promote gene activation independent of any effect on histone acetylation, and can be essential for leukemia stem cell to self-renewal and malignant transformation (4, 61).

### Tip60

#### Tip60 is Required for an Oncogene-Induced DNA-Damage Response

*Tip60* regulates several transcription factors, which can promote or suppress carcinogenesis (e.g., p53 and Myc). *Tip60* regulates DNA-damage response (DDR) signaling induced by oncogenes, which can prevent cancer progression. Loss of one allele of *Tip60* inhibited Myc-induced DDR but had no effect on DDR in normal B cells. In the *Tip60* heterozygous knockout mice, *Tip60* inhibits Myc-induced lymphomagenesis at the pre-tumoral stage. The mono-allelic loss of *Tip60* occurs often in lymphomas with concomitant reduction in mRNA levels; this often coexists with p53 mutations and is related to the disease grade. Thus, *Tip60* functions as a tumor suppressor in a haplo-insufficient manner, and *Tip60* is essential for controlling Myc-induced DDR in cancer cells (62).

#### Tip60 is Involved in c-Myb-Driven Leukemogenesis

*TIP60* interacts with c-Myb, which requires the transactivation domain of c-Myb and the HAT domain of *TIP60*. Coexpression of *TIP60* impairs c-Myb-induced transcription activation. *TIP60* can bind to the promoters of c-Myb target genes, which is dependent on c-Myb. Furthermore, c-Myb interacts with HDAC1 and HDAC2, which are associated with *TIP60* and cause transcriptional repression. *TIP60* negatively regulates the transcription activity of c-Myb through interacting with HDACs in human hematopoietic cells. Consistently, knockdown of *Tip60* increased the expression level of c-Myc. It has been found that the expression level of *Tip60* is much (~60%) lower in the patients with AML. These findings suggest that *TIP60* regulates the function of c-Myb and that dysregulated *TIP60* could be involved in c-Myb-driven leukemogenesis (63).

### MOZ/MORF

#### The MOZ-TIF2 Fusion Protein is Associated with AML

MOZ–TIF2 is associated with AML chromosomal abnormalities at inv(8)(p11q13). MOZ–TIF2 contains the CBP interaction domain (CID) of TIF2 and the HAT domains of MOZ and TIF2 (64–67). In a murine bone marrow transplant assay, MOZ–TIF2 causes AML that could be serially transplanted (67). It has been found that the interaction between MOZ–TIF2 and CBP through the CID domain and the C2HC nucleosome recognition motif in MOZ are essential for transformation (68). MOZ–TIF2 dominantly inhibits the transcription activity of CBP-dependent activation (e.g., p53 and nuclear receptors), which requires the CID domain. The nuclear localization of MOZ–TIF2 is abnormal, which is dependent on the MOZ portion of this fusion protein. CBP expression is decreased in the cells expressing MOZ–TIF2, which results in the depletion of CBP from PML bodies. The critical characteristics of MOZ–TIF2 are to disrupt the normal activity of CBP/CBP-dependent activators in acute myeloid leukemia (69). MOZ–TIF2 binds to the promoter of RARβ2, leading to the dissociation of CBP/p300, the abnormal histone acetylation and the downregulation of RARβ2. MOZ–TIF2 was recruited to AML1 target promoters and upregulated transcription mediated by AML1. Both MOZ and MOZ–TIF2 were found to co-localize with AML1 (70). MOZ–TIF2 impaired retinoic acid-mediated transcription activation of C/EBPβ/CD11b, and inhibited nuclear receptor-induced gene activation through aberrant recruitment of CBP, which required both the MOZ and TIF2 domains of this fusion protein (71).

In a transgenic zebrafish model, spi1 promoter-driven MOZ–TIF2 expression induced the development of AML in 2 of 180 embryos expressing MYST3/NCOA2, in which kidney invasion by myeloid blast cells was observed (72). MOZ–TIF2 interacted with PU.1 to stimulate the expression of M-CSFR, and PU.1 is required for the establishment and maintenance of LSCs by MOZ–TIF2. Loss of CSF1R impairs MOZ–TIF2-induced leuekmogenesis in mouse models and CSF1R inhibitors delay the development of MOZ–TIF2-induced AML (73). MOZ–TIF2 can cooperate with FLT3–ITD to transform hematopoietic cells. STAT5 signaling is required for MOZ–TIF2-induced leukemogenesis. Deletion of STAT5 led to the differentiation of MOZ–TIF2-transduced fetal liver cells, and these cells lost their replating ability. The recipient mice transplanted with Stat5^−/−^ MOZ–TIF2 leukemia cells have longer latency and incomplete penetrance. STAT5 is essential for the self-renewal of leukemia stem cells in MOZ–TIF2 driven leukemia (74). Overexpression of HOXA9, HOXA10, and MEIS1 was observed in AML patients with MOZ fusions. MOZ–TIF2 forms a stable complex with bromodomain-PHD finger protein 1 (BRPF1), and MOZ–TIF2/BRPF1 associate with Hox genes in the MOZ–TIF2 driven leukemia cells. BRPF1 depletion disrupts the localization of MOZ on the Hox genes, which led to the inhibition of MOZ–TIF2-induced transformation. Moreover, mutant MOZ–TIF2 lacking HAT activity could not deregulate HOX genes or initiate AML. Thus, MOZ–TIF2/BRPF1 induces leukemogenesis through the upregulation of HOX genes, which is regulated by MOZ-dependent histone acetylation (75).

#### MOZ–CBP and MOZ–p300 are Generated by Chromosomal Translocations in AML

The recurrent translocation *t*(8;16)(p11;p13) leads to the MOZ–CBP fusion gene, which contains the MOZ finger motifs and HAT domain and a mostly intact CBP (76). This *t*(8;16)(p11;p13) translocation occurs in the M5 subtype AML, which is characterized by erythrophagocytosis and a poor prognosis. The CBP–MOZ mRNA is not in-frame, which suggested that MOZ–CBP is the critical fusion for the initiation of leukemia (77, 78). MOZ, CBP, and MOZ–CBP are all able to acetylate the transcription factor AML1. The level of MOZ–AML1 complex upregulates when M1 myeloid cells differentiate into macrophages/monocytes. This finding suggests that the MOZ–AML1 complex could have important functions in the differentiation of myeloid cells. MOZ–CBP inhibits the differentiation of M1 myeloid cells, and could induce the development of leukemia through impairing AML1-induced transcription activation (79). MOZ–CBP cooperates with steroid receptor co-activator-1 to activate transcription, and the CBP portion of MOZ–CBP is required for the transcription activity of this fusion protein. It has been found that the interaction between MOZ–CBP and NF-κB could also have critical functions in leukemogenesis (80). MOZ–CBP inhibits p53-mediated transcription, and the impairment of MOZ/p53-induced transcription contributes to the development of leukemia (81).

The translocation *t*(8;22)(p11;q13) in acute myeloid leukemia generates the fusion gene MOZ–p300, and the MOZ zinc finger/HAT domain are fused to a mostly intact p300. Thus, MOZ–p300 has two HAT domains from MOZ and p300 portions, and may play an important role in the development of leukemia through deregulation of histone acetylation (82). It has been found that MOZ is fused with an unknown partner at 2p23 in one patient with tMDS. Therefore, MOZ might lead to the abnormal histone acetylation and promote the pathogenesis of myeloid malignancies (83).

### GCN5/PCAF

The *t*(1;19) translocation was found in pediatric pre-B cell ALL, which leads to the fusion of E2A and PBX1 and the generation of a *E2A–PBX1* fusion protein. *E2A–PBX1* is able to induce the transformation of hematopoietic cells. It has been found that SPT3–TAFII31–GCN5L acetylase (STAGA) and its HAT subunit *GCN5* directly binds to the E2A portion of *E2A–PBX1*. *GCN5* can acetylate and stabilize the *E2A–PBX1* fusion protein (23). AML1/MDS1/EVI1 (AME), a transcription repressor generated by translocation *t*(3;21) in human leukemia, binds to P/CAF and *GCN5* through two binding sites, with one of the binding sites being in the Runt domain. *GCN5* and P/CAF are able to acetylate AME, and either P/CAF or *GCN5* can cooperate with AME to impair the repression of *AML1*-dependent transcriptional activation (84, 85).

### The important role of p53 acetylation in hematological malignancies

The transcription factor p53 was the first non-histone substrate discovered to be acetylated by HATs (5). Levels of p53 acetylation are associated with the activation and stabilization of p53 (86–93); acetylation of p53 also stimulates its sequence-specific DNA-binding (94–97). The seven different lysine (K164, K305, K370, K372, K373, K381, and K382) in the C-terminus of p53 are acetylated by CBP and PCAF (98). Acetylation of p53 is crucial for the recruitment of CBP/p300 to the promoters of its target genes. CBP and p300 are able to promote p53-mediated transcription activation (99). TIP60/hMOF acetylates p53 at position 120 (100, 101), which can be induced by DNA damage or oncogenic stress-mediated p19ARF activation (102). Mutation of p53 lysine 120 to arginine inhibited p53-induced transcription activation. p53 can also be acetylated at one lysine outside its C-terminus, which is critical for the activation of the proapoptotic genes PUMA and BAX. In the p53 acetylation-deficient knockin mouse, the expression of p53 target genes is decreased after DNA damage (103). The deletion of the lysine residue at position 164 and other acetylation sites in p53 blocked p53-mediated transactivation of p21 and inhibition of cell growth (104). These findings suggest that p53 acetylation may play a critical role in the pathogenesis of hematological malignancies.

### Third generation HDAC inhibitors for the therapy of hematological malignancies

The FDA approved drug, Beleodaq, is used for the therapy of patients with peripheral T cell lymphomas (PTCL), an aggressive disease, which accounts for ~15% of all non-Hodgkin lymphomas. Beleodaq inhibits HDAC and it is the third drug to receive FDA approval for PTCL. In the trial that led to the FDA approval, Beleodaq’s overall response rates were comparable to those of Folotyn and Istodax; 10.8% of patients experienced a complete response and 15% had a partial response. The response rate was even higher in patients with angioimmunoblastic T cell lymphoma, which suggests that targeting acetylation is a promising therapeutic strategy for the therapy of hematological malignancies.

## The Potential Therapeutic Effects of Histone Acetyltransferase Inhibitor in Hematopoiesis

### The HAT inhibitor, garcinol, induces the expansion of hematopoietic stem/progenitor cells

The HAT inhibitor, Garcinol, is derived from plants. It has been identified as a stimulator of human HSPCs expansion in the screening of natural products. During a 7-day culture of CD34^+^CD38^−^ HSCs or CD34^+^ HSPCs, Garcinol was able to induce the expansion of HSPCs, and this ability is associated with its inhibitory effect on HATs. The derivatives of Garcinol, which can expand HSPCs, are also able to inhibit HAT activity and histone acetylation. Altogether, the Garcinol effects suggest that targeting HATs could be a promising strategy for expanding HSPCs (105).

### Inhibition of p300 impairs antitumor immunity

Foxp3^+^ Treg cells can not only regulate immune homeostasis/autoimmunity but also limit the immune response of hosts to tumors. Thus, targeting Foxp3^+^ Treg cells could be a promising strategy to improve antitumor immunity. Conditional deletion or pharmacological inhibition of p300 was able to increase T cell receptor-induced apoptosis in Foxp3^+^ Treg cells and abrogate the suppressive functions of Treg cells. Inhibition of p300 can also impair the induction of peripheral Treg cells and tumor growth in the immunocompetent mouse model. Collectively, p300 is critical for the homeostasis and function of Foxp3^+^ Treg cells, and targeting p300 could be a new approach for cancer immunotherapy (54).

### The p300 inhibitor, C646, acts selectively on *t*(8;21) leukemia Cells

The HAT p300 can enhance the self-renewal ability of leukemia stem cells through acetylating AE and activating the target genes of AE, which indicated that p300 could be a promising drug target for *t*(8;21) leukemia. C646, a selective and competitive p300 inhibitor, can inhibit the proliferation and colony formation of *t*(8;21) leukemia cells, and induce apoptosis and G1 phase cell cycle arrest in *t*(8;21) leukemia cells and primary cells isolated from patients with *t*(8;21) leukemia. C646 does not significantly affect the normal HSPCs mobilized by GCSF. In particular, AML1^−^ETO^+^ AML cells are more sensitive to C646 compared with AML1^−^ETO^−^ AML cells. The growth inhibition of AML1^−^ETO^+^ leukemia cells induced by C646 are associated with the decreased acetylation of histone H3 and downregulation of Bcl2/C-kit, suggesting that C646 could be a promising drug candidate for the treatment of AML1^−^ETO^+^ leukemia (4, 106).

### The HAT inhibitor, epigallocatechin-3-gallate, inhibits B cell transformation

Epigallocatechin-3-gallate (EGCG) has been found as a HAT inhibitor in natural compound screening. EGCG can block p300-mediated acetylation of p65, impairing its translocation to the nucleus, and it can upregulate the amount of IκBα in the cytoplasm, thus inhibiting NF-κB activity in several ways, and decreasing the expression of NF-κB target genes. EGCG impairs B cell transformation by EBV, perhaps via suppression of NF-κB acetylation (107), and it can inhibit the binding of p300 to the IL-6 promoter and block cytokine gene expression. Thus, EGCG could be a potential therapy for B cell malignancies.

### Leukemia and lymphoma cell lines are sensitive to p300 inhibitor L002

p300 plays an important role in signal transduction pathways that promote the proliferation and survival of malignant cells. Therefore, p300 represents a promising drug target for hematological malignancies, and libraries of compounds have been screened for p300 inhibitors. One candidate, L002, inhibits p300 *in vitro*, with an IC50 of ~2 μM. L002 can block histone acetylation and p53 acetylation, and can inhibit the activation of STAT3. Biochemical testing of a series of related compounds revealed functional groups that may impact the inhibitory potency of L002 against p300. Interestingly, these analogs show inhibitory activities against CBP, PCAF, and GCN5, but against several other acetyltransferases (KAT5, KAT6B, and KAT7), HDACs and HMTases. Among the NCI-60 panel of cancer cell lines, leukemia, and lymphoma cell lines were extremely sensitive to L002. Thus, this new acetyltransferase inhibitor, L002, is a potential anticancer agent (108).

### Anacardic acid derivatives inhibit PCAF and induce apoptosis in chronic myeloid leukemia cells

The different acetylation of proteins correlates with the development of BCR–ABL-positive leukemia. A derivative of anacardic acid – small molecule MG153, which is developed to have stronger HAT inhibitory ability, is a potent inhibitor of PCAF. The inhibition of PCAF decreases proliferation and induces apoptosis, which correlates with loss of the mitochondrial membrane potential and DNA fragmentation. Importantly, cells expressing BCR–ABL are more sensitive to PCAF inhibition compared to parental cells without BCR–ABL. Moreover, inhibition of PCAF in BCR–ABL-expressing cells breaks their resistance to DNA damage-induced cell death. Targeting the PCAF alone or in combination with DNA-damaging drugs shows cytotoxic effects and should be considered as a prospective therapeutic strategy in chronic myeloid leukemia (CML) cells. Moreover, anacardic acid derivative MG153 is a valuable agent and further studies validating its therapeutic relevance should be performed (109).

### GCN5 inhibitors have anti-leukemia effects

The a-methylene-g-butyrolactone 3 (MB-3) is a cell-permeable inhibitor against GCN5, and is able to decrease the levels of histone H3 acetylation and non-histone substrate (a-tubulin) acetylation. GCN5 acetylates E2A–PBX1, and MB-3 reduces the levels of E2A–PBX1 acetylation and E2A–PBX1 protein in a dose-dependent manner. RCH–ACV cells are derived from the bone marrow cells of a patient with pre-B cell acute lymphoid leukemia, and has *t*(1;19) translocation, which generates the *E2A–PBX*1 fusion gene. E2A–PBX1 acetylation was inhibited by MB-3, and the level of E2A–PBX1 and GCN5 protein was decreased when RCH–ACV cells were treated with MB-3. The E2A–PBX1 half-life was shorter in the cells treated with MB-3, indicating GCN5-dependent acetylation can affect the stability of E2A–PBX1 protein. A reduction in Wnt16, an E2A–PBX1 target gene were also observed in RCH–ACV cells cultured with MB-3, indicating the importance for the pathogenesis of *t*(1;19)-positive pre-B cell leukemia. Additionally, the expression of E2A–PBX1, E2A, and Wnt16 were significantly decreased in the primary *t*(1;19) pre-B ALL cells treated with MB-3. MB-3 does not affect the expression of Pol II or Tubulin, which suggests that MB-3 can destabilize certain proteins probably through the inhibition of GCN5-dependent histone or non-histone substrate acetylation. These findings indicate that GCN5 inhibitors have potential value as therapeutic agents for ALL (23). Some recently identified GCN5 inhibitors, such as (thiazol-2-yl)hydrizones (110, 111), might also be able to target hematological malignancies.

## Conclusions

Lysine acetylation acetylation occurs not only at the histone tails but also in the non-histone proteins. LATs are catalytic subunits of multiprotein complexes, whose biochemical and molecular characterization have yielded much important information about the function and regulation of acetyltransferase activity. Importantly, HATs have the catalytic/non-catalytic and histone/non-histone effects on the hematopoietic cells, which confer HAT the ability to control a variety of cellular events in normal and malignant hematopoiesis. Genetic approaches are very useful to study how protein acetylation controls a variety of cellular events in normal and malignant hematopoiesis. The study on the gene knockout mouse models shows that p300 and CBP play distinct roles in hematopoiesis; GCN5 but not PCAF is essential in early embryonic development. Thus, such an *in vivo* approach will generate the new findings on the role and mechanism of LATs. One major finding in the mechanism study on the function of protein acetylation is that bromodomain can bind to the acetylated lysine. Since a lot of proteins have bromodomain, it would be interesting to understand whether all bromodomains can recognize the acetylated lysine and how bromodomains specifically recognize the acetylated lysine. MOZ, MORF, p300, and CBP are involved in the leukemia-associated chromosomal translocation, which can generate leukemogenic fusion genes. Thus, LATs have critical functions in the pathogenesis of hematological malignancies. The direct involvement of LATs in hematological malignancies indicates that compounds with the ability to regulate the activity of LATs are the potential drugs for the treatment of hematological malignancies. Thus, the studies on the function and mechanism of histone/non-histone proteins acetylation will not only shed new light on how lysine acetylation controls a variety of cellular events in normal and malignant hematopoiesis but also provide critical insights into the development of new therapeutic strategies for the therapy of hematological malignancies.

## Conflict of Interest Statement

The authors declare that the research was conducted in the absence of any commercial or financial relationships that could be construed as a potential conflict of interest.
